# Vectorial synchrony: A method for quantifying directional alignment of audience movements using three-axis acceleration data

**DOI:** 10.1016/j.mex.2026.104184

**Published:** 2026-09-26

**Authors:** Ryota Nomura, Shijun Wang

**Affiliations:** Faculty of Human Sciences, Waseda University, 2-579-15 Mikajima, Tokorozawa, Saitama, 359-1192, Japan

**Keywords:** Accelerometry, Audience response, Collective movement, Directional alignment, Live performance, Time-series analysis

## Abstract

Collective audience movements at live performances are difficult to quantify unobtrusively and on a large scale. We propose vectorial synchrony, a method that reduces three-axis acceleration data from multiple participants to a scalar measure of directional alignment at each time point. The method uses smartphone accelerometers and a measurement application to collect audience movements without using dedicated sensing devices. Its computational complexity scales linearly with the number of valid participants, supporting the processing of large groups. We evaluated the method in two field settings: a men’s cheerleading performance and a public viewing of a music video. Audience arm movements were recorded, and vectorial synchrony was calculated in both settings. In the validation test, surrogate-data testing identified significant temporal increases and decreases in synchrony corresponding to changes in the performance. These results demonstrate that smartphone accelerometers can be used to measure collective movement over time and that vectorial synchrony provides a scalar signal for performance analysis and interactive control.

• Smartphone accelerometers enable the measurement of audience movement without requiring dedicated sensing devices.
• Vectorial synchrony summarizes directional alignment as a scalar time series.
• Field experiments demonstrate the method under live performance conditions.

## Highlights

• Smartphone accelerometry enables field sensing of audience arm movements.
• Vectorial synchrony quantifies directional alignment of audience arm movements.
• The algorithm scales linearly with the number of valid observations.
• Periods of significantly high or low vectorial synchrony in audience movements corresponded to the song's structure.

## Graphical abstract

**Figure:**
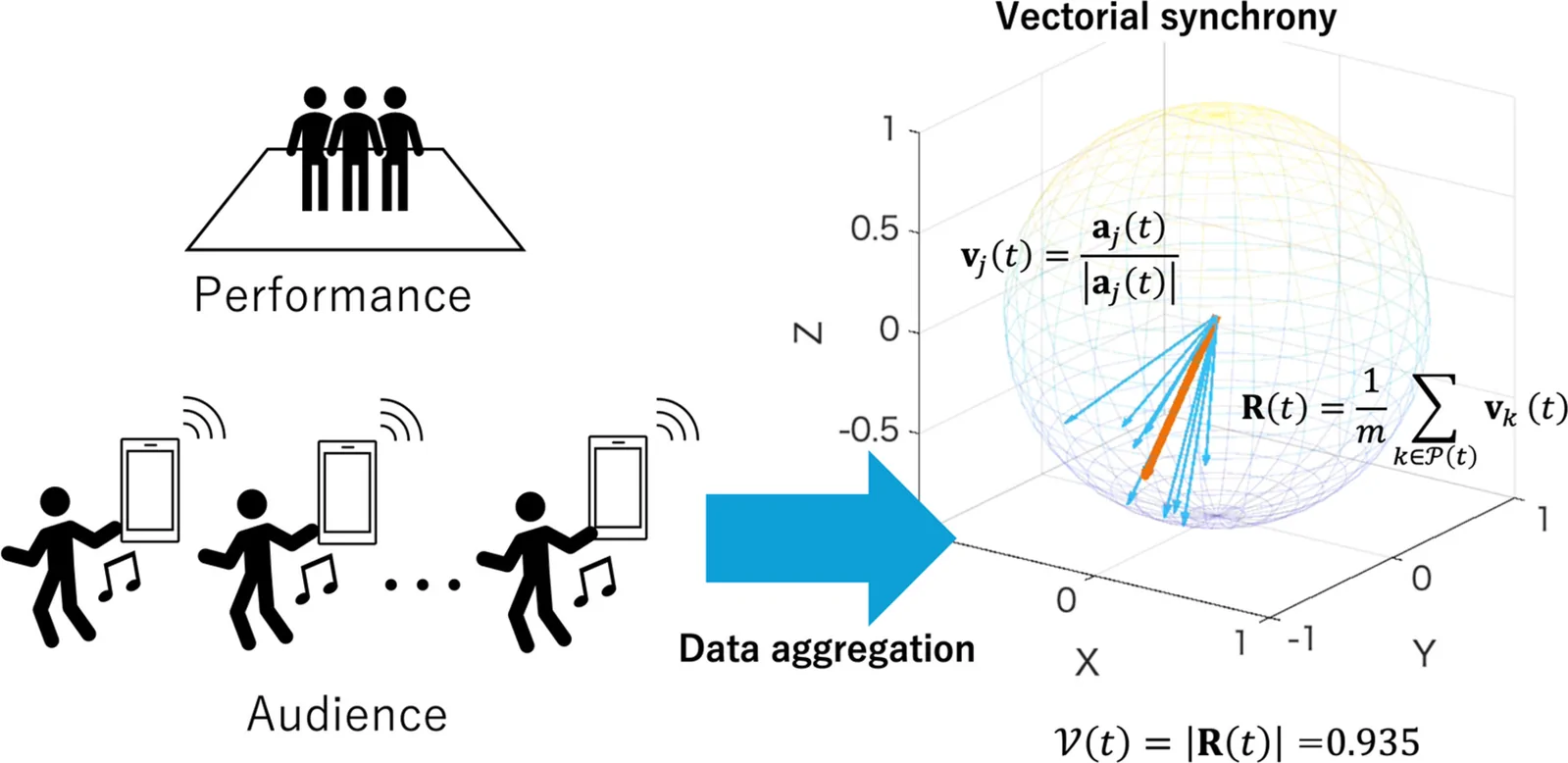

**Specifications table**

**Table:** 

**Subject area**	Psychology
**More specific subject area**	Quantitative performance and audience research; multi-participant movement analysis
**Name of your method**	Vectorial synchrony
**Name and reference of original method**	Spherical generalization of the Kuramoto order parameter described by Lipton, Mirollo, and Strogatz (2021). The present method applies the resultant-vector norm to empirical three-axis acceleration vectors at each sample.
**Resource availability**	The method requires a smartphone with a three-axis accelerometer. The calculation can be implemented using any software that supports basic vector operations, and the equations and processing procedure are provided in this article. The measurement application used for data collection was developed for this study and is not publicly available. No specialized hardware is required.

## Background

A live performance is a collective event in which audience members exhibit synchronized behavioral and physiological responses. As such synchrony becomes better characterized, it is increasingly being considered not only as an object of study [[1], [2], [3], [4]] but also as information that may support performance analysis and creative development. This growing interest has increased the need to quantify synchrony in collective audience behavior over time.

Current approaches to sensing collective behavior typically rely on audio or video recordings, or on pairwise analyses of physiological or motion signals. Although these methods are effective in controlled settings, they have practical limitations when applied to large crowds. Video- and audio-based systems require dedicated infrastructure and raise privacy concerns. Furthermore, pairwise synchrony measures based on correlation perform poorly with a large number of participants and require long temporal windows to achieve statistical stability. These limitations hinder their real-time deployment and restrict their applicability in uncontrolled environments.

In settings such as stadiums, concert halls, and public events, human movement emerges continuously, reflecting both individual actions and group dynamics. In such environments, the measurements must be unobtrusive and scalable to several thousand participants. They must also be robust to noise and missing data and computationally efficient to support real-time processing.

Therefore, in this study, we propose a recording system that quantifies the collective synchrony of spectators’ body movements using inertial sensor data. Rather than inferring individual intentions or psychological states, our approach focuses on extracting a low-dimensional, time-resolved measure of group-level synchrony from high-dimensional motion signals obtained from spectators. Using the built-in three-axis accelerometers of the participants’ smartphones, this method enables unobtrusive data acquisition in real-world settings without requiring specialized hardware or external infrastructure.

Specifically, we introduce vectorial synchrony, V(t), derived from an extension of the Kuramoto order parameter applied to the empirical three-dimensional acceleration vectors. At each time point, the acceleration signals from multiple participants are normalized and combined into a single scalar value that reflects the extent to which the crowd is aligned in terms of direction. The computational cost of the proposed index scales linearly with the number of valid participants, making it suitable for real-time sensing even in large-scale deployments.

We validated our data collection and calculation method through field experiments conducted in live performance settings, including a cheerleading event and a public viewing of a music concert. Using smartphone-based inertial data collected *in situ*, we demonstrate that the synchrony index robustly captures temporal fluctuations in collective movement patterns under realistic conditions. The proposed method provides a practical sensing primitive for large-scale human-centric sensor systems.

## Method details

### Data collection

To capture the extent to which the crowd is aligned in terms of direction, we developed a system that uses the built-in accelerometers of smartphones owned by audience members (iPhones in our experiments) to measure bodily movements without using dedicated penlight-shaped measurement devices. Fig. 1 shows the system configuration. A measurement application running on each audience member’s iPhone was launched. The three-axis acceleration data obtained from each iPhone (Fig. 2) were automatically transmitted and stored on the server when the application was closed. The stored data contained no personally identifiable information, including UDIDs. Thus, the participants were fully anonymized after data collection, and individual data could not be linked to their sources.

**Fig. 1:**
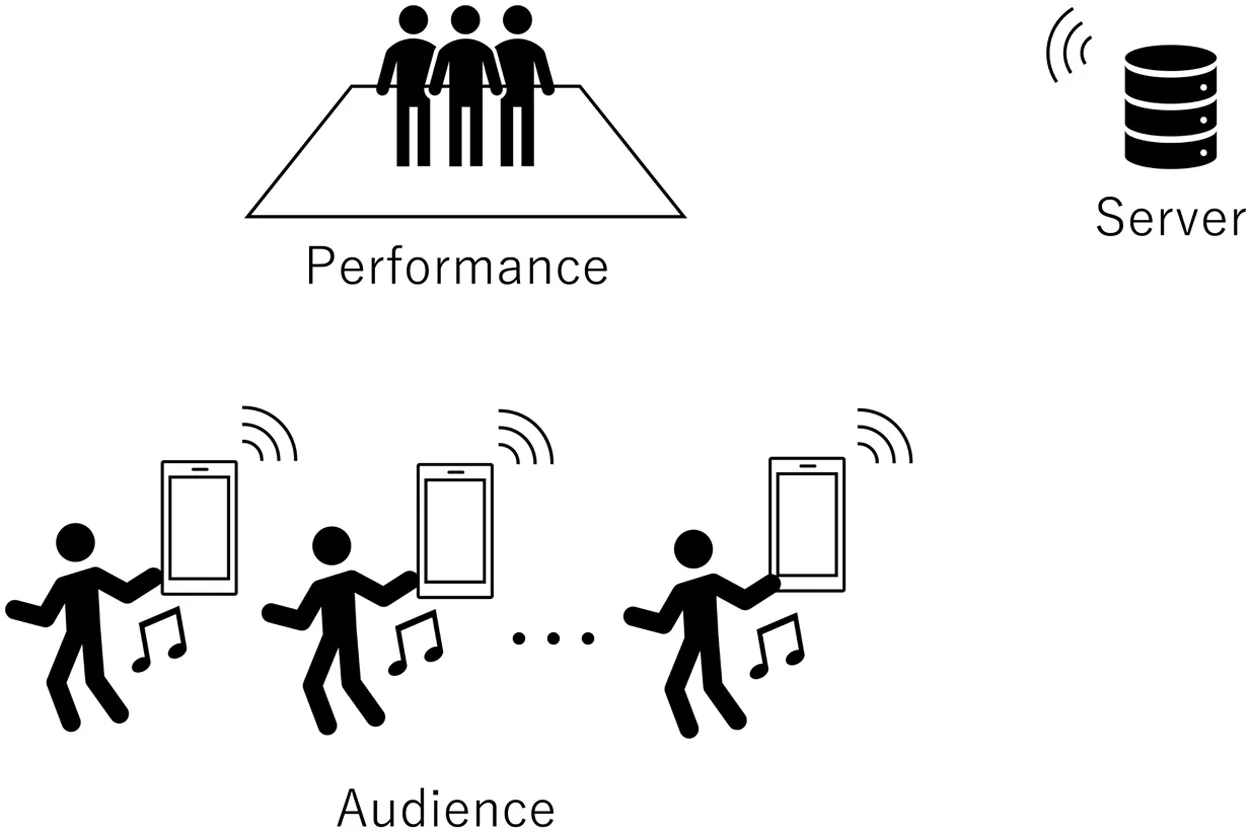
Overview of the measurement application system.

**Fig. 2:**
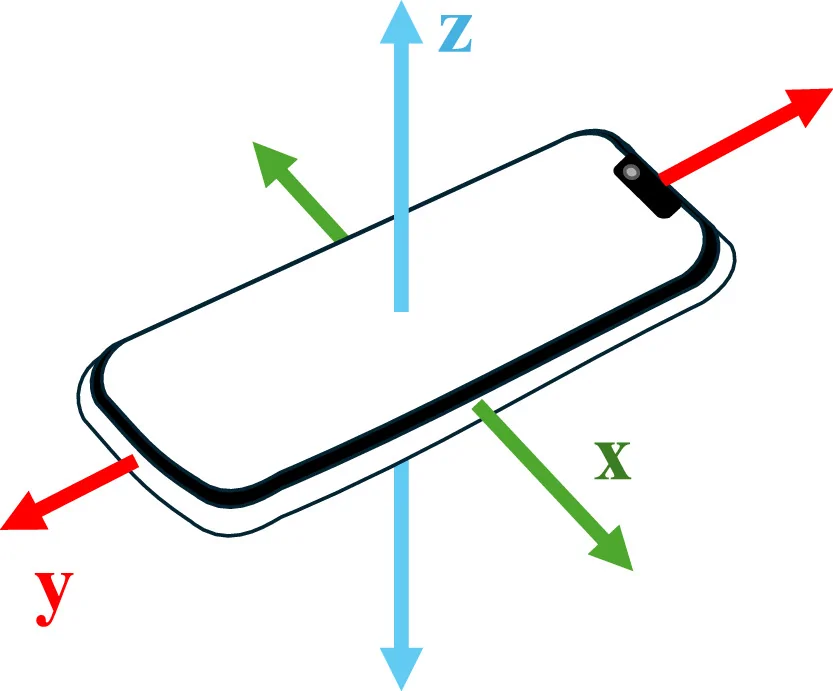
Three axes of the accelerometer used for sensing.

The application was designed for use at sporting events and live music performances. When a participant pressed the Start button, the measurement began, and an external light was turned on, producing a penlight-like effect. In both experiments, no specific phone orientation was prescribed. Participants generally held the iPhone upright (with its y-axis approximately vertical), with the screen facing themselves and the outward-facing light directed toward the stage or video screen. Real-time data transmission can be implemented in future versions of the system for use in performance production. However, in the present study, data were transmitted to the server when the application was closed because our primary objective was to determine whether the collected data could be used to calculate the synchrony index.

### Synchrony index

In this study, we propose an index for quantifying the degree of synchrony in audience members’ bodily movements using three-axis acceleration data obtained from sensor-equipped devices such as smartphones. To represent synchrony in these data as a single scalar, we propose vectorial synchrony, V(t), which corresponds to a three-dimensional Kuramoto order parameter [5]. In this framework, the state of each oscillator is represented as a point on the unit sphere $S^{d-1}$ embedded in the d-dimensional Euclidean space $R^{d}$. The degree of collective synchrony is characterized by the distance between the centroid of these points and the origin. Although the conventional Kuramoto model assumes all-to-all coupling among the oscillators, the proposed index is intended only to quantify synchrony and does not assume coupling.

Let x, y, and z denote the three accelerometer axes (Fig. 2). At time t, the acceleration measured along these axes by the device of the audience member j is represented by

$$a_{j}\left(t\right)=\left[a_{x_{j}}\left(t\right),\;a_{y_{j}}\left(t\right),\;a_{z_{j}}\left(t\right)\right].$$

The magnitude of this acceleration vector is defined as

$$\left|a_{j}\left(t\right)\right|=\sqrt{a_{x_{j}}{\left(t\right)}^{2}+a_{y_{j}}{\left(t\right)}^{2}+a_{z_{j}}{\left(t\right)}^{2}}.$$

To retain only the directional information, we normalized the vector and mapped it onto a point on the unit sphere:

$$v_{j}\left(t\right)=\frac{a_{j}\left(t\right)}{\left|a_{j}\left(t\right)\right|}.$$

If $|a_{j}\left(t\right)|=0$ or any component was missing, the state of the audience member j at that time was treated as undefined and excluded from subsequent analyses (see the ideal-sphere illustration). Let P(t) denote the set of participants with valid observations at time t, and let |P(t)|=m denote the number of such participants. We then define the vector summarizing the collective behavior as follows:

$$R\left(t\right)=\frac{1}{m}\sum_{k\in P\left(t\right)}v_{k}\left(t\right).$$

This vector is the arithmetic mean of the points on the unit sphere calculated in Euclidean space and represents the mean direction of the group. Therefore, we use its norm as the vectorial synchrony index, V(t)=|R(t)|. By definition, V(t) ranges from 0 to 1. It equals 1 only when all $v_{k}\left(t\right)$ point in the same direction and is approximately 0 when the vector directions are isotropically distributed over the sphere [5].

Missing values are common in empirical data. Because only valid data are used at each time point, the index can robustly characterize overall synchrony, even when a small number of observations are missing. Moreover, the index can be calculated after the first sampling interval: 0.1 s after the start of the measurement at 10 Hz and 1 s after the start of the measurement at 1 Hz. In addition, the computational complexity of the index is O(m), which scales linearly with the number of valid participants m. Therefore, even for audiences with tens of thousands of participants, the computational cost is expected to increase linearly, thereby enabling real-time processing on a general-purpose computer (Fig. 3).

**Fig. 3:**
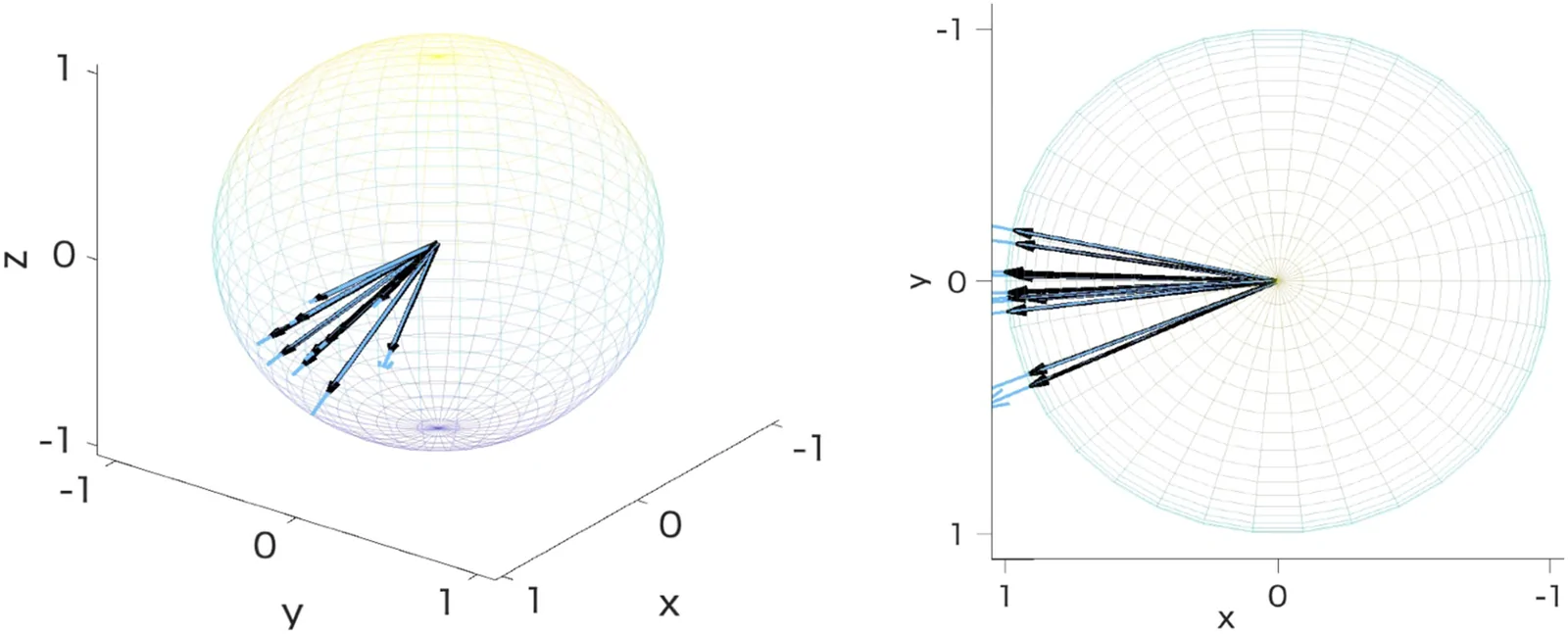
Conceptual illustration of the synchrony index. The acceleration vector obtained from an audience member (light blue) is mapped to a unit vector (black) on the unit sphere. The figure shows the case V(t)=0.98.

## Method validation

### Experiment overview

We conducted validation experiments in two field settings: live sports viewing and music listening. These experiments examined whether the proposed application (Audience_C™) and synchrony index could measure synchrony in audience arm movements while participants remained focused on the performance of athletes or performers. In Experiment 1, we evaluated whether the measurement application, which had been tested under laboratory conditions, could be used in an actual theater. We then calculated the proposed synchrony index using the collected data. In Experiment 2, we refined the experimental conditions based on the results of Experiment 1. Both experiments involved new analyses of data originally collected for undergraduate theses submitted to the School of Human Sciences at Waseda University in the academic years 2024 and 2025, respectively. The synchrony index proposed in this study was newly applied to these datasets.

### Experiment 1: men’s cheerleading

#### Objective

As the first field experiment, Experiment 1 examined whether the measurement application operated as intended and whether the data were properly transmitted to the server. We also confirmed that the synchrony index proposed in this study could be applied to the collected data.

#### Overview and participants

Experiment 1 was conducted in the Main Hall of the Nerima Culture Center. The performance was *SHOCKERS STAGE 2024*, organized by SHOCKERS, the men’s cheerleading team at Waseda University. Approximately 300 spectators attended the performance, of whom 33 students and fans aged 18−27 participated in the experiment. Although all participants performed arm-swing movements while watching the performance, many recordings could not be obtained because the iPhones automatically entered sleep mode when there was no input, such as screen touches. After excluding these records, data from up to 12 participants with continuous and simultaneous recordings were used for the subsequent analyses.

#### Methods and procedure

Participants held an iPhone in their preferred hand and launched the measurement application immediately before the performance. Arm movements were recorded as three-axis acceleration data at intervals of approximately 0.1 s. Each participant launched the application in response to a signal from the experimenter immediately before the performance began and stopped it when the stage lights went dark at the end of the performance. Measurements were obtained for three routines: the opening, standard, and ending sequences, each lasting approximately 5 min. The results for the standard sequence are reported because this routine had the largest number of participants with continuous recordings. For the analysis, the timelines of all participants were aligned according to the elapsed time from the beginning of the performance. Time stamps were synchronized using the iPhone's built-in Network Time Protocol. We then calculated V(t) using each participant’s three-axis acceleration vector averaged over nonoverlapping 1-s time windows.

#### Results and discussion

Examples of the techniques performed during the performance are shown in Fig. 4. The V(t) values calculated from the arm movement data of the 12 participants with continuous recordings are shown in the right panel of Fig. 5. The figure indicates a high degree of synchrony at approximately 51 and 125 s after the start of the performance. These scenes correspond to the end of the opening sequence and the beginning of the tumbling sequence, respectively. The mean acceleration magnitude of the audience members’ arm movements, $|a_{j}\left(t\right)|$, was higher during the tumbling sequence (Fig. 4, right) than during the toss sequence (Fig. 4, left) or the stunt sequence (Fig. 4, center). These results suggest that performance elements associated with increased excitement may enhance audience synchrony in addition to increasing movement intensity.

**Fig. 4:**
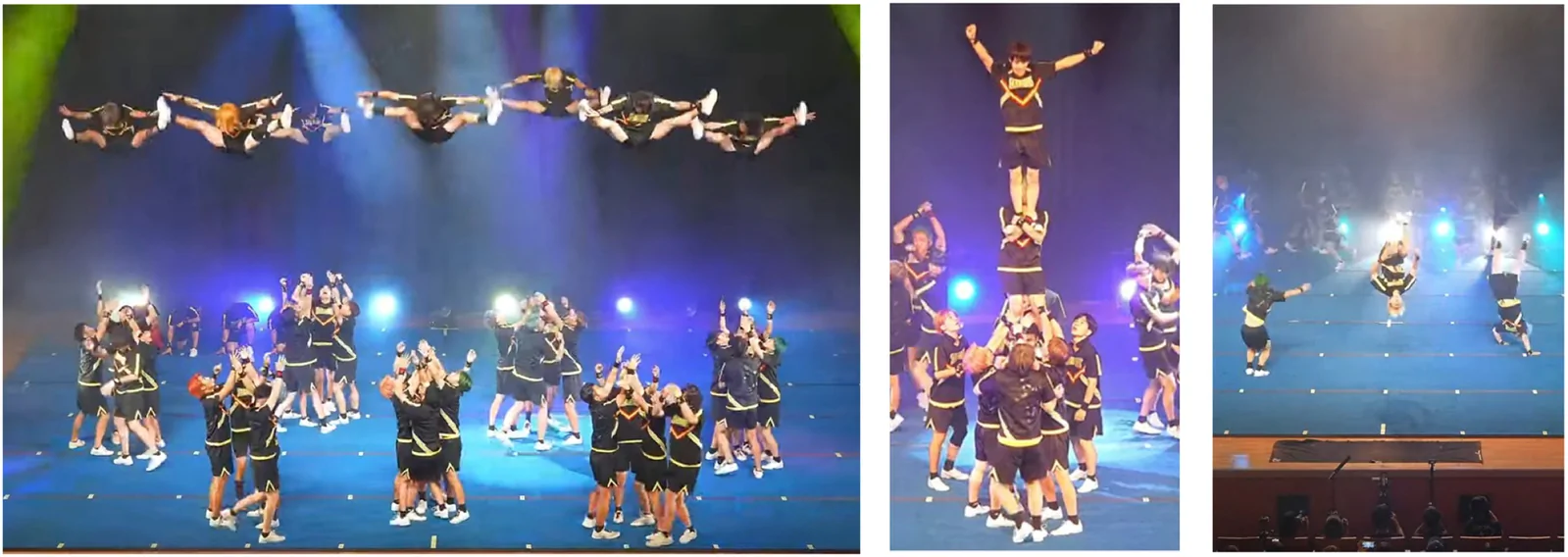
Examples of cheerleading techniques: toss (left), three-level stunt (center), and tumbling (right).

**Fig. 5:**
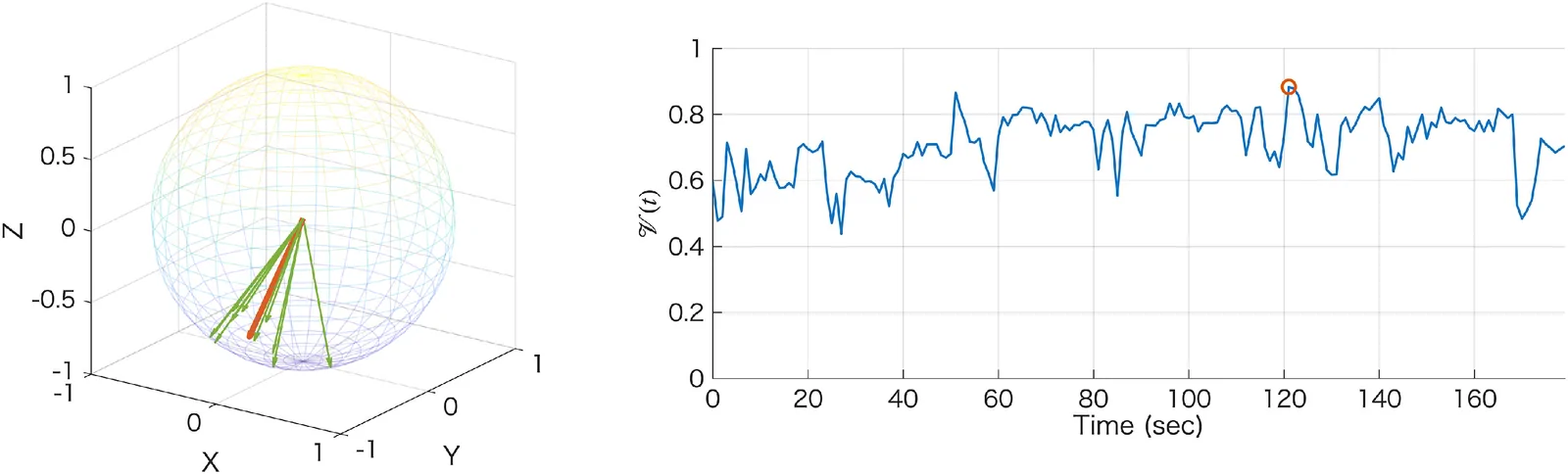
Vectorial synchrony, V(t),during the cheerleading routine. The left panel shows the vector configuration used to calculate V(t), and the right panel shows the temporal variation in V(t) from the beginning of the routine. The left panel shows the vector configuration at the time points marked by circles in the right panel.

### Experiment 2: public viewing

#### Objective

Thus, Experiment 1 demonstrated that three-axis acceleration data could be recorded in an actual theater and that the synchrony index could be calculated. However, several issues remained unresolved. First, many participants could not be measured until the end of the performance because of the iPhone sleep-mode settings. This issue requires improvement through advance explanations and instructions regarding the device settings. In addition, even when the calculated V(t) was relatively high, it was not possible to determine whether the value was statistically significant. Based on these issues, Experiment 2 included modified measurement instructions and introduced a statistical test.

#### Overview and participants

Experiment 2 was conducted at Stella Hall in Komoro, Nagano, Japan. The target performance was a music video approximately 4 min 30 s long that had been publicly released on YouTube. The audience consisted of 25 people, of whom 11 participated in the iPhone-based measurement: 10 students and 1 faculty members (4 men and seven women), with a mean age of 23.1 years and a standard deviation of 6.64 years.

#### Methods and procedure

Before the experiment, the iPhone setting that automatically activated sleep mode was disabled. As in Experiment 1, the measurement application recorded the three-axis acceleration vector $a_{j}\left(t\right)$ of each audience member j at each time point t, at intervals of approximately 0.1 s.

Participants launched the application before the video began. On the experimenter’s cue, they performed arm movements sequentially in seat order, allowing their seating positions to be identified. The music video was then shown in full on the screen in front of the audience in a public-viewing setting. While watching the video, participants freely moved their arms while holding the iPhone in their preferred hand, and these movements were recorded by the application. Participants stopped the application at the end of the performance, at which point the data were transmitted to the server.

All data recorded during the video viewing were considered for analysis. However, data from one of the 11 participants could not be used because of a measurement problem. Consequently, data from 10 participants were used in the final analysis.

#### Significance testing using random-shuffling surrogate data

We calculated V(t) and applied a surrogate-data method based on random shuffling to assess the significance of the value at each time point. Specifically, for each audience member, the time indices of $a_{j}\left(t\right)$ were randomly shuffled. This procedure preserved the mean and standard deviation while removing temporal correlations. We generated 1,000 surrogate datasets containing data from the 10 participants and calculated V(t) for each dataset.

Using these surrogate values as the reference distribution, the observed value of V(t) was considered significant if it exceeded the upper 2.5% boundary or fell below the lower 2.5% boundary of the surrogate distribution.

#### Results and discussion

First, we calculated the synchrony index V(t) from the audience members’ arm-movement data and assessed its statistical significance. To examine the relationship between V(t) and movement intensity, we then calculated the mean value of $|a_{j}\left(t\right)|$ across the audience members. The comparison is shown in Fig. 6.

**Fig. 6:**
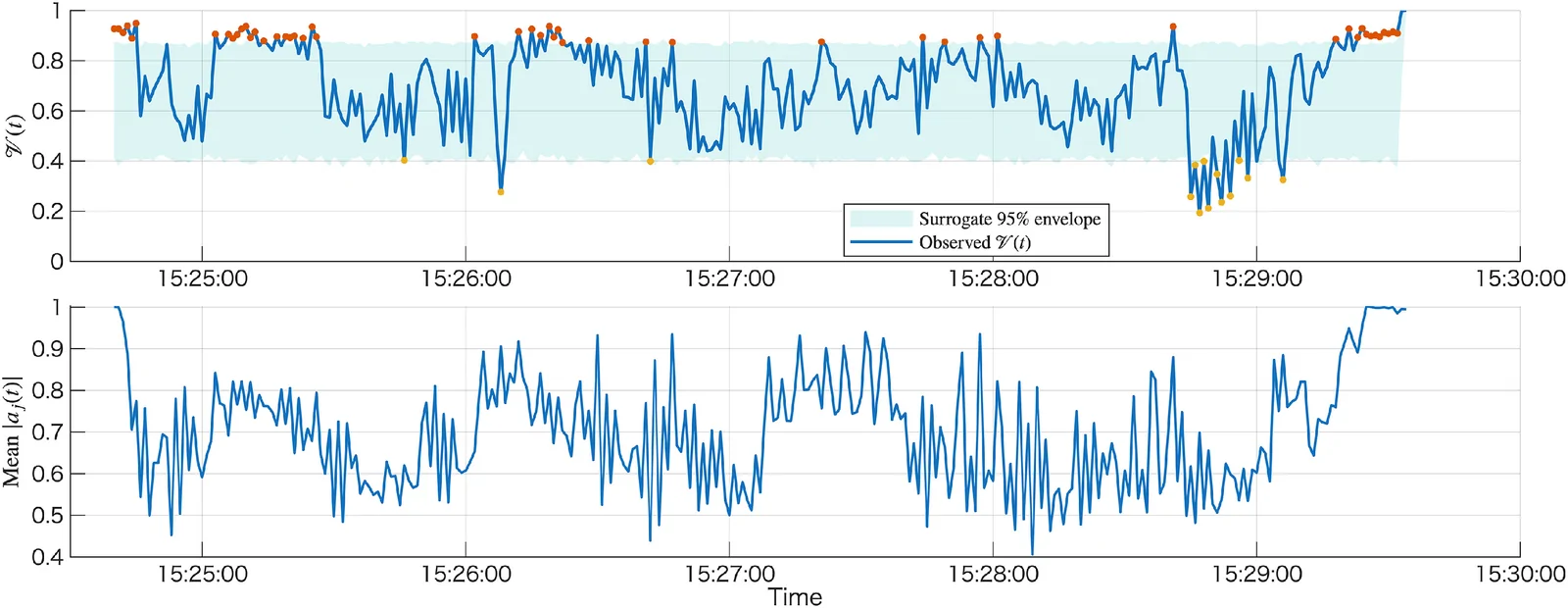
Temporal variation in V(t) (top) and $|a_{j}\left(t\right)|$ (bottom) during the performance. The light cyan band in the top panel indicates the 95% confidence interval calculated using the surrogate method.

As shown in the figure, V(t) was significantly higher during (a) 15:25:03–15:25:26, approximately 23 s after the start of the video, when singing began; (b) 15:26:12–15:26:28, corresponding to 1:32–1:48 during the verse; and (c) 15:28:40, corresponding to 4:01 and the last phrase of the chorus. In contrast, V(t) was significantly lower during (d) 15:28:45–15:29:06, corresponding to 4:05, and the period immediately after the chorus through the outro, when a wide shot of the audience was shown.

These results indicate that the synchrony index was higher, particularly around the vocal onset and offset of the singer. The vectors were concentrated in a common direction during these periods (Fig. 7a). However, no statistically significant V(t)value was observed during the chorus, indicating that synchrony was not consistently high throughout this section. In addition, the mean acceleration was not consistently high during periods of high synchrony. In the live-viewing setting, audience members could continuously perform arm movements, resulting in movement intensity above a certain level. Therefore, the results suggest that it is difficult to identify audience excitement from mean acceleration alone, which has been the primary measure used in previous approaches.

**Fig. 7:**
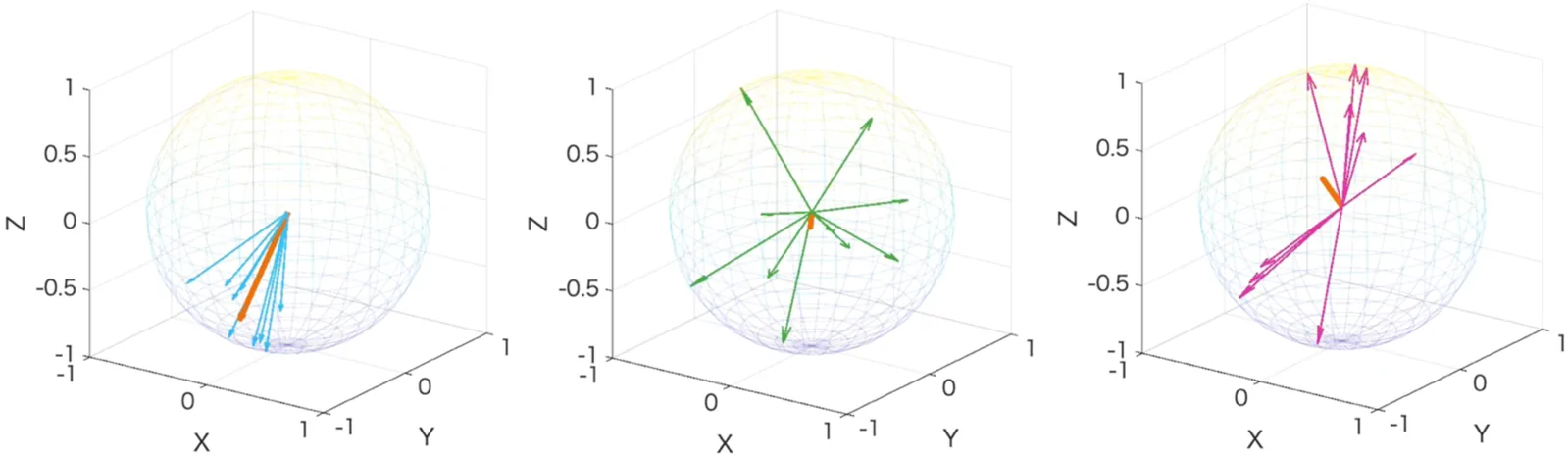
Relationship between the audience’s arm-swing vectors and the synchrony index. (a) High synchrony (V(t)=0.935): the last phrase of the chorus (15:28:41). (b) Low synchrony (V(t)=0.194): the outro (15:28:47). (c) Low synchrony (V(t)=0.276): a scene with a bimodal distribution of vector directions (15:26:08).

The period in (d), during which synchrony significantly decreased, corresponded to a scene in which the lyrics had ended, the instrumental outro was being played, and footage of the audience was projected. The projected footage showed a large audience moving their arms rhythmically in time with the performance. In contrast, the participants in Experiment 2 who watched this footage were observed to make small oscillatory movements along the x-axis or slow movements along the y-axis (Fig. 7b). Therefore, the synchrony index decreased because the movement vectors were distributed across different directions.

#### Overall discussion

Across Experiments 1 and 2, the measurement system successfully recorded audience arm movements, although the instructions provided by the experimenter regarding settings such as sleep mode and screen brightness require further refinement. In the music public-viewing data, the synchrony index was significantly higher or lower at time points corresponding to specific performance events. These results suggest that the index has some degree of validity.

Although the data obtained in the validation experiments were limited in quantity, the synchrony index can be readily applied to large-scale data. If the arm movements of up to tens of thousands of audience members were measured in real time at a stadium or concert venue, the server load could pose a technical challenge. However, the transmitted data are text data, and such an operation appears practically feasible given current communication capabilities. In addition, the computational complexity of the synchrony index is O(m). Thus, the computational cost increases only linearly as the number of participants reaches tens of thousands and is expected to remain low. Future studies should examine whether arm movements can be measured for many participants and whether the synchrony index can be calculated in real time.

From a theoretical perspective, the proposed application and synchrony index can be used not only to describe collective excitement but also to investigate its generation mechanisms. Because the proposed index was designed solely to quantify the degree of synchrony, it did not assume all-to-all coupling among audience members. However, in real collective excitement, synchrony among audience members is likely to involve both the performance as a common input from the performers and interactions among audience members. A possible future application is to estimate local interactions among audience members using individual-level data obtained by the measurement application system.

Because the proposed synchrony index is represented by a single scalar value at each time point, it is suitable for applications in which lighting and sound effects are adjusted according to quantified synchrony in audience movements. Potential applications include controlling parameters such as sound volume, light intensity, and the degree of randomness according to the synchrony index. Bidirectional interactions between performers and audiences have been suggested to enhance audience immersion. Such applications could promote synchrony and collective emotional experiences synergistically.

## Limitations

The proposed method has several limitations. In addition to cases in which the synchrony index decreases because the vector directions are uniformly distributed, we also identified cases in which the index decreases because the audience’s arm movements form two groups in opposite phases. As shown in Fig. 7(c), at 15:26:08, the vectors were divided mainly along the positive and negative directions of the z-axis, corresponding to the front–back direction when the iPhone was held upright. A decrease in the synchrony index under these conditions is a consequence of its definition. However, from the perspective of quantifying the alignment of audience movements, a formulation incorporating symmetry may be appropriate in some applications so that anti-phase synchronization also produces a high synchrony value.

A practical issue in this context is the correction of gravitational acceleration. When the iPhone is stationary, gravitational acceleration is aligned with the negative direction of the y-axis (Fig. 2). Consequently, V(t)≈1 can be obtained even in the absence of arm movements, as observed during the first 5 s of the music in Fig. 6. Measurement accuracy could be improved by detecting the orientation of the iPhone using its built-in gyroscope and correcting for gravitational acceleration according to the detected orientation. When gyroscope orientation data are unavailable, zero-phase high-pass filtering of each acceleration axis (e.g., with a 0.3–0.5 Hz Butterworth filter) before vector normalization may attenuate the gravity component. Because phone orientations were not standardized, differences in tilt or rotation could also alter vectorial synchrony. Accordingly, when this application is used in theaters, the measurement setup and participant instructions should specify a common phone orientation.

## Ethics statements

Informed consent for the two experiments was obtained from all participants before participation. The experimental procedures were approved by the Ethics Review Committee on Research with Human Subjects of Waseda University under the following protocols: “The Impact of Cheerleading Choreography on Audience Enthusiasm” (2023-294) and “Research on the Relationship between the Experience of Watching Live Music and Seating Position” (2024-608).

## CRediT authorship contribution statement

**Ryota Nomura:** Conceptualization, Methodology, Software, Formal analysis, Writing – original draft. **Shijun Wang:** Writing – review & editing.

## Declaration of competing interest

The authors declare that they have no known competing financial interests or personal relationships that could have appeared to influence the work reported in this paper.

## Data Availability

Data will be made available on request.

## Appendix Supplementary materials

application 1
