# Supplementary material for: Vectorial synchrony: A method for quantifying directional alignment of audience movements using three-axis acceleration data
Source: MethodsX. 2026 Sep 26;17:104184. doi: 10.1016/j.mex.2026.104184 (PMC13656952; doi:10.1016/j.mex.2026.104184)
Supplement: Supplementary file 1 [file mmc1.docx]

Supplementary Algorithm

**Article:** Vectorial synchrony: A method for quantifying directional alignment of audience movements using three-axis acceleration data

**Algorithm S1. Calculation of vectorial synchrony and the pointwise 95% surrogate envelope**

**Input:** Time-stamped three-axis acceleration data from the included participants.

**Parameters:** Number of surrogates B = 1,000; norm threshold eps = 10^-9.

**Code**

```
For each participant j and nonoverlapping 1-s window t:
    a[j,t] <- (mean_valid{ax[j,s] : s in t},
               mean_valid{ay[j,s] : s in t},
               mean_valid{az[j,s] : s in t})
    if all axes are finite and norm(a[j,t]) > eps:
        v[j,t] <- a[j,t] / norm(a[j,t])
    else: v[j,t] <- missing
For each t: V[t] <- norm(mean of valid v[j,t] across j)
            (undefined if no valid vectors exist)
For b = 1, ..., B:
    Independently for each j, permute complete valid v[j,t]
    across valid t; retain the missing positions
    Recalculate V_b[t] for every t
For each t: [L[t], U[t]] <- 2.5th and 97.5th percentiles
                         of {V_b[t] : b = 1, ..., B}
```

mean_valid averages available samples and is missing if no samples are available.
